# The Gut Microbiome Profile in Obesity: A Systematic Review

**DOI:** 10.1155/2018/4095789

**Published:** 2018-03-22

**Authors:** Olga Castaner, Albert Goday, Yong-Moon Park, Seung-Hwan Lee, Faidon Magkos, Sue-Anne Toh Ee Shiow, Helmut Schröder

**Affiliations:** ^1^Cardiovascular Risk and Nutrition Research Group, Hospital del Mar Research Institute (IMIM), Barcelona, Spain; ^2^CIBER de Fisiopatología de la Obesidad y Nutrición (CIBEROBN), Santiago de Compostela, Spain; ^3^Department of Endocrinology and Nutrition, Hospital del Mar Research Institute, Barcelona, Spain; ^4^Department of Medicine, University Autònoma de Barcelona and Universitat Pompeu Fabra, Barcelona, Spain; ^5^Epidemiology Branch, National Institute of Environmental Health Sciences, National Institutes of Health, Research Triangle Park, NC, USA; ^6^Division of Endocrinology & Metabolism, Department of Internal Medicine, Seoul St. Mary's Hospital, College of Medicine, The Catholic University of Korea, Seoul, Republic of Korea; ^7^Department of Physiology, Yong Loo Lin School of Medicine, National University of Singapore (NUS) and Clinical Nutrition Research Centre (CNRC), Singapore Institute for Clinical Sciences (SICS), Agency for Science, Technology and Research (A^∗^STAR), Singapore; ^8^Yong Loo Lin School of Medicine, National University of Singapore, Singapore; ^9^Division of Endocrinology, Department of Medicine, National University Health System, Singapore; ^10^Regional Health System Planning and Development, Singapore; ^11^CIBER de Epidemiologia y Salud Pública (CIBERESP), Madrid, Spain

## Abstract

Gut microbiome has been identified in the past decade as an important factor involved in obesity, but the magnitude of its contribution to obesity and its related comorbidities is still uncertain. Among the vast quantity of factors attributed to obesity, environmental, dietary, lifestyle, genetic, and others, the microbiome has aroused curiosity, and the scientific community has published many original articles. Most of the studies related to microbiome and obesity have been reported based on the associations between microbiota and obesity, and the in-depth study of the mechanisms related has been studied mainly in rodents and exceptionally in humans. Due to the quantity and diverse information published, the need of reviews is mandatory to recapitulate the relevant achievements. In this systematic review, we provide an overview of the current evidence on the association between intestinal microbiota and obesity. Additionally, we analyze the effects of an extreme weight loss intervention such as bariatric surgery on gut microbiota. The review is divided into 2 sections: first, the association of obesity and related metabolic disorders with different gut microbiome profiles, including metagenomics studies, and second, changes on gut microbiome after an extreme weight loss intervention such as bariatric surgery.

## 1. Introduction

Obesity is known to be a major public health problem that affects more than 1.9 billion adults, which means 39% of adults are considered obesity and overweight [1]. Estimations from the noncommunicable diseases (NCD) collaboration reported that if these trends continue, by 2025, global obesity prevalence will reach 18% in men and surpass 21% in women; severe obesity will surpass 6% in men and 9% in women [2]. Obesity is associated with multiple pathologies, among them cardiovascular diseases, metabolic syndrome, and cancer [3].

The latest public statements report the attributable deaths to obesity. The Global BMI Mortality Collaboration reported that mortality increased with body mass index (BMI) approximately in a log-linear manner and that the associations of both overweight and obesity with higher all-cause mortality were broadly consistent in four continents [4].

Another troubling factor is the increase of childhood obesity, which is known to be a risk factor for obesity in adults [5].

Gut microbiota has captured our attention in the last decade as an element that directly affects our health or disease status. In particular, it has been implicated in the aetiology of obesity [6]. In fact, the composition of gut microbiota seems to play a role concerning obesity.

Gut microbiota is considered as an assortment of microorganisms that inhabit the gastrointestinal tract. The composition of this microbial community depends on the host, but it can also be modified by exogenous and endogenous events [7].

With regard to the host, these bacteria are symbiotic, and play an important role in physiological processes, for example, in digestion, or they can intervene in the metabolism, as they can increase energy production from the diet and take part in the regulation of the fatty acid tissue composition [8]. The different bacteria can also induce low-grade inflammation. All these processes are involved in obesity and metabolic disorders.

With the introduction of human whole metagenome studies, the associations of microbiota and disease were plausible and many have been encountered.

It is known that most of the human's populations microbiota is composed by 5 phyla Bacteroidetes, Firmicutes, Actinobacteria, Proteobacteria, and Verrucomicrobia [9] being Bacteroidetes and Firmicutes around 90% of the total bacterial species [9, 10].

The composition of the bacterial diversity seems to change between lean and obese, increasing the number of Firmicutes to the detriment of Bacteroidetes [9, 11–13] in obese patients and also in type 2 diabetes, which is pathology in close relationship to obesity. But some recent studies have found controversial results [9, 11–15]: studies of association between microbiota profiles and different phenotypes or body mass index have adventured the positive and negative associations among the different phyla that populate the intestines. The searches of mechanistic pathways involved in the contribution of microbiota to obesity or vice versa have been subject for many animal studies [10]. An interesting finding was that obesity-resistant germ-free mice become obese and increase their energy harvest and caloric uptake after receiving a microbiota transplant [16].

The new era of sequencers has widely unlocked the acquirement of information. Sequencing of specific regions of 16S or 18S ribosomal genes allows the identification of organisms and their relative abundance in purified DNA [17]. Thus, the sequencing of gut microbiota with 16S rRNA made an inflexion point in the knowledge and relationship of the microbial diversity with the different physiopathological states that intervene in health and disease, allowing the observation of the behavior of the different bacterial phyla, and genus, links with different phenotypes, different types of diets, and obesity in particular [18]. Furthermore, the development of high throughput DNA sequencing of shotgun genomic libraries to assess the microbiome (collection of all genes in the microbial genomes), such as the Human Microbiome Project, is aiming to contribute to the assessment of the diversity and relative abundance of our commensal microbiota at different sites of our organism and to the understanding of their role on human health and disease [19].

Bariatric interventions have been implemented as a solution to extreme situations of weight gain in the past decades. The techniques have improved with two main variants: the Roux-en-Y gastric bypass (RYGB) and the laparoscopic sleeve gastrectomy (LSG).

RYGB and LSG procedures modify the anatomy of the gastrointestinal system, which modulates nutrient transit and impacts gut physiology. They are known to produce a long-term reduction in body weight and to decrease blood glucose levels, both of which are relevant to obesity-related type 2 diabetes and cardiovascular disease. Nevertheless, the mechanisms implicated in the metabolic improvements associated with bariatric surgery are still challenging. [20]

Some studies have pointed to the effect of surgery on the microbiota diversity as a partial contribution to the resolution of the metabolic status of these patients [21].

This review focuses on the current evidence of the associations between the microbiota profile and the individuals' phenotypes and on the effect of bariatric surgery on gut microbiota.

## 2. Methods

### 2.1. Eligibility Criteria

We assessed observational human studies or clinical trials that evaluated the gut microbiota of individuals who suffered from obesity. Obesity was defined by body mass index (BMI). We also selected observational studies of extreme weight loss interventions, such as bariatric surgery, but did not include dietary interventions, because there is a lack of homogeneity, and many reviews have already focused on this theme.

We selected the MeSH terms “Obesity” AND “Microbiota,” with the following filters: language: English, French, and Spanish; publication date: 5 years from August 20, 2017. The search was performed in MEDLINE accessed by PubMed. All of them were screened based on the title and abstract.

Other possible articles were screened and searched on the reference lists of the selected articles. With the reading of the title or title and the abstract, we selected observational studies and clinical trials, and systematic reviews, in humans.

## 3. Results

### 3.1. Literature Search

General characteristics of the studies are as follows: From the 570 articles retrieved from the search, 83 articles were selected based on the title and abstract to be read in depth. Finally, 15 studies were included and are described in Tables 1 and 2. Eleven articles assessed differences in the microbiota profile between obese and lean; most of these studies were performed on adults, but 3 of them were performed on children. Four articles assessed the effects on microbiota of bariatric surgery. 68 were discarded including the reviews which we read but did not include in the study except if they were systematic (see Figure 1 of Supplementary Materials).

All the studies had a baseline assessment of the gut microbiota, and the changes in microbial composition were assessed as outcomes.

The 11 studies compared a baseline assessment of gut microbiota from different individual phenotypes whereas the 4 studies which involved bariatric surgery compared the baseline assessment with the change after surgery at different time points within each subject.

Most of the studies were performed in Europe (75%), America (33%), and Asia (25%).

Several molecular biology techniques were used to assess the characterization of the gut microbiome: denaturing gradient gel electrophoresis (DGGE), fluorescence in situ hybridization probes (FISH), metagenomics shotgun sequencing, and characterization of the 16S rRNA genes in a sample, and groups specific real-time polymerase chain reaction (PCR) are some of the assays. Some studies have made a step forward and published results of functional analysis of the gut microbiota and metabolome. We have focused on the analysis of the diversity of microbiota among BMI.

With regard to the time of publication, more than 50% were published in the last 2 years 2015–2017.

#### 3.1.1. The Association of Obesity and Related Metabolic Disorders with Different Gut Microbiome Profiles

Among the latest studies on microbiota profile between lean and obese patients, several studies searched for differential gut microbiota signatures associated with obesity. We found 11 interesting studies, which compare microbiota in individuals with different BMIs.

The first reported study observed that the bacterial diversity was significantly greater in obese subjects compared with nonobese subjects [22]. Next-generation sequencing revealed that obese and nonobese subjects had different gut microbiota compositions and that certain bacterial species were significantly associated with each group. This study also agrees that the ratio Firmicutes/Bacteroidetes (F/B) was higher in obese subjects and also overweight subjects (BMI > 25) [22].

In the case of the individuals who suffered from obesity, there was a strong association with the following bacterial species from the Firmicutes phylum: *Blautia hydrogenotorophica*, *Coprococcus catus*, *Eubacterium ventriosum*, *Ruminococcus bromii*, and *Ruminococcus obeum*. On the other hand, in the lean individuals, there was a larger proportion of the Bacteroidetes phylum species *Bacteroides faecichinchillae* and *Bacteroides thetaiotaomicron* and also Firmicutes *Blautia wexlerae*, *Clostridium bolteae*, and *Flavonifractor plautii*. In agreement with these results, another study with a focused approach also observed that Firmicutes (*L. reuteri*) was associated with obesity and another genus *Methanobrevibacter smithii* was decreased. In this case, the authors focused directly on targeting Firmicutes, Bacteroidetes, *Methanobrevibacter smithii*, *Lactococcus lactis*, and *Bifidobacterium animalis*, by quantitative PCR and cultures [23].

Furthermore, a third party was introduced with gender, in a study with the aim at observing differences in microbiota between genders, but reported that the differences in gender could be influenced by BMI [24]. They observed that the F/B ratio changed along with BMI, as it had been described before [22], and also by gender. The F/B ratio tended to increase with BMI up to >33 and decreased surprisingly when BMI was >33. In men, Bacteroidetes genus decreased when the BMI increased, but no changes were observed in women.

The methodologies differ between these studies. In our sequence of studies, the three assessments were performed in fecal samples of adult individuals who suffered from obesity comparing with normal weight adult individuals. The two first studies were performed by metabarcoding 16S rRNA [22, 24], and the third one used a different methodology with a focused approach on PCR and cultures [23].

Two of these studies were performed in Europe and the other one in Asia [22]; this fact is important because BMI categories differ between Asia and Europe.

Another two publications analyzed the upper gastrointestinal microbial diversity [25, 26] compared to the above-mentioned studies that assessed microbiota from fecal samples. None of these two studies of microbiota from the upper gastrointestinal tract and duodenum found differences in the microbiota diversity in terms of phyla, which may suggest that the impact of obesity might affect the lower portions of the gastrointestinal tract.

The analysis of the upper gastrointestinal microbial diversity did not find association among the bacterial community with obesity. Alpha diversity was not associated with obesity but beta diversity was. The microbiome was characterized using the 16S rRNA gene DNA microarray (the HOMIM array), which uses 16S rRNA-based oligonucleotide probes printed on glass slides. They also used another approach to search for diversity in the community and found that BMI was not associated with the bacterial community diversity as assessed by alpha diversity in their models after adjusting for multiple potential confounders. However, BMI was significantly associated with the variation in the community composition, as assessed by multiple beta-diversity parameters. As a limitation in this study, the microarray was only semiquantitative and contained a limited number of bacterial species. As a microarray based on the 16S rRNA gene, this assay did not produce data that could be used to determine categories of bacterial functions.

Another study performed in upper gastrointestinal tract, studying the microbiota of duodenum [26], also reported that there were no differences between obese and lean among the microbiota phyla. The only differences found were in the relative abundance of aerobic and anaerobic, being the obese population, presenting a higher proportion of anaerobic genera, mostly *Veillonella*, *Bulleidia*, and *Oribacterium*.

A very interesting study [27] aimed at confirming some previous published results of studies of considerable magnitude, such as the HMP (Human Microbiome Project from the NIH) and the MetaHIT project, and also at comparing them with two previous studies of high reputation [11, 28]. The researchers analyzed all the results together and could not find an association, so no difference was found between obese and lean individuals in their relative abundance of Bacteroidetes or Firmicutes. And interestingly, it was found that the variation in the relative abundance of Firmicutes and Bacteroidetes was much larger among studies than between lean and individuals who suffered from obesity within any study. The relevance of this specific article is that it gives scientific justification that there are some real statistically proved discrepancies among the studies and that MetaHIT and HMP not only do not recapitulate the findings observed but even go in the opposite direction. So, at this point, no significant association between BMI and taxonomic composition at the phylum level could be found.

Other studies have been performed in twin pairs, which provide more information about the heritability of microbiota: one of the studies analyzed fecal samples of healthy monozygotic (MZ) twin pairs which were discordant in weight and compared them with other concordant BMI twin pairs [29]. Their results demonstrated that within-pair similarity was a dominant factor independent of acquired obesity. Another study [30] that wanted to assess which specific taxa within the gut microbiome are heritable and to what extent and how do heritable microbes relate to host BMI. They compared fecal samples from twins and found a greater similarity of microbiota within twin pairs compared to unrelated individuals.

The most heritable taxon overall was the family Christensenellaceae (Firmicutes phylum), which associates with a low BMI. The family Christensenellaceae was significantly enriched in subjects with a lean BMI (<25) compared to those with an obese BMI (>30).

Within the three most dominant bacterial families, from the Firmicutes phylum and families Ruminococcaceae and Lachnospiraceae, there was a significantly greater similarity for MZ twins compared to dizygotic (DZ) twins, in contrast with the Bacteroidaceae family, in which MZ and DZ twins had similar pairwise diversity. Therefore, Firmicutes seems to have more heritability.

Three studies have focused their attention on gut microbiota association to obesity in children. Two of them were performed in Central and South America in children up to 11 years old. Another study performed in youngsters aged 13–16 was performed in Asia.

A Mexican study [31] in 190 children did not find statistical differences between phyla. Despite that, several genera and families, from the Firmicutes phylum as well as some Enterobacteriaceae, increased in overweight and obese children: the genus *Faecalibacterium* sp., the family Lachnospiraceae, and the genus *Roseburia* sp. A decrease in the genus *Succinivibrio* sp., the genus *Erwinia* sp. from the Proteobacteria phylum, and the genus *Oscillospira* sp. from the Firmicutes phylum was found.

The genus *Blautia* sp., the genus *Coprococcus* sp., and the family Enterobacteriaceae from the Firmicutes phylum were clearly increased in the overweight phenotype. Another study in 30 obese children (3–11 years) found that there were high concentrations of *B*. *fragilis* group and *Lactobacillus* sp. in obese and overweight children when compared with the lean ones, with the fecal concentrations positively correlated with BMI [32]. Furthermore, a negative correlation between BMI and *Bifidobacterium* spp. was observed.

In contrast, another study in Korean adolescents did not find any significant differences in the Bacteroidetes, Firmicutes, and Proteobacteria populations in samples from normal and obese adolescents at the phylum level [33]. However, there was a marked difference in the average proportions of *Bacteroides* and *Prevotella* between normal and obese samples at the genus level. This trend persisted at the family level. A significant association was found between the compositions of several bacterial taxa and child obesity, with the proportion of *Bacteroides* highest in normal children compared to those who suffered from obesity.

With regard to the metabolically healthy obese subject, defined as those subjects which have normalized levels of the parameters that are used to define metabolic syndrome (blood pressure, HDL cholesterol levels, glycaemia, and visceral fat), there is no literature characterizing the microbiota profile of these subjects. Such findings would be interesting to understand whether the microbial diversity has a metabolic role in individuals who suffered from obesity.

#### 3.1.2. Bariatric Surgery Weight Loss Impact on Gut Microbiota

We have found 4 articles in the last 5 years to look for the effect of bariatric surgery on the microbiota profile. All four studies analyze the results comparing with a baseline before the intervention and have a follow-up of 6 months to 1 year. Two of them studied the changes after RYGB surgery, another one LSG, and another one included both types of surgery.

Palleja et al. found that after RYGB surgery, the microbial diversity increased and this diversity was maintained one year after surgery [36]. Another study that included the two types of surgery only found an increased diversity in the RYGB group whether there were no differences between individuals who suffered from obesity, and the LSG group, which lead us to think that maybe the type of surgery and not the weight loss is playing a role in the diversity.

Opposite to this assumption, another study in which BS was LSG, compared to a diet, showed that both interventions resulted in changes of the Bacteroidetes/Firmicutes ratio but with an inverse relationship between the main phyla [34]. While LSG increased Bacteroidetes and decreased Firmicutes, the dietary intervention resulted in reduced Bacteroidetes in favour of Firmicutes. The Bacteroidetes/Firmicutes ratio decreased following dietary intervention, whereas in the LSG group, this ratio increased. In the LSG group, the number of Bacteroidetes showed a negative correlation with body weight while Firmicutes numbers were positively correlated with body.

Finally, another study that only recruited patients with a RYGB found that four of the top seven high abundance phyla were decreased in postoperative samples, including Firmicutes (from 47.2 to 34.2%), Bacteroidetes (from 46.9 to 44.7%), Actinobacteria (from 1.7 to 1.2%), and Cyanobacteria (from 0.10 to 0.06%). However, the ratios of Bacteroidetes/Firmicutes shifted from 0.99 to 1.31, showing an apparent increase.

## 4. Discussion

This review intends to recapitulate the information obtained in the last 5 years on the association of gut microbiota with obesity and an extreme weight loss intervention. One of the important points of discussion is whether obesity is associated with more or less microbiota diversity and whether the ratio F/B is increased with obesity.

Still some controversial data have been published in the past 5 years. Whereas previous relevant studies [28] had found lower microbiota diversity in those individuals who suffered from obesity, compared to lean individuals, our review depicts that most of the studies did not find differences concerning bacterial diversity in unrelated population studying the upper GI tract [25], weight discordant twins [35], or children [31, 33]. One of the reasons for not finding associations in the upper gastrointestinal tract might be also due to the less abundant number of bacteria which exponentially increase from the proximal to the distal gastrointestinal tract and so being the colon where most of the bacteria are harboured [17]. Also in the case of the studies on children [31–33], the gut microbiota starts at birth, reaches maximum diversity at adolescence, and remains stable until later stages of life [17], or studies age span was between 3 and 16 years old, which would justify the diversity of the results. Also, bariatric surgery in any of its varieties increased gut microbial diversity [36, 37].

Among the presented studies, one study observed an increase in alpha diversity which was also related to an increase in the ratio F/B [22]. In concordance with this finding, it seemed to be described more abundance of Firmicutes in obese subjects even with different types of methodologies [23], and also this ratio seemed to be higher in women when BMI was increased [24] or in a specific family within the phylum [23]. These observations follow the fact that after BS the ratio decreases with a decrease of Firmicutes and an increase of Bacteroidetes [29, 34] and as it had be previously reported [16].

Among the limitations of the studies, the new and growing advance in methodologies such as next-generation sequencing has arouse many different possibilities in terms of laboratory work and data management and software, as mentioned above in a comparative study [27] which clearly shows how the intervariability between studies is greater than the difference between lean and individuals who suffered from obesity.

## 5. Conclusion

This review systematically assessed studies of association between obesity and microbial diversity of the gastrointestinal tract and bariatric surgery interventions in obese and overweight patients. Obesity is associated with different profiles of gut microbiota, but studies seem not to find enough consistency on the results, most probably because it can be influenced by several factors, among them the different methodologies and growing data management knowledge. Also, we review that bariatric surgery intervention for weight loss impacts the gut microbiota composition.

Further trials and the evolution of this shotgun sequencing data management are needed to draw conclusions about the role of microbial diversity in obesity.

## Figures and Tables

**Table 1:** Lean/obese clinical trials.

Study identification	Description	*N*	Population description	Outcomes
Kasai et al. 2015 [22]	Cross-sectional study	56 (10)	Japanese population: 23 BMI < 20 kg/m^2^ and 33 BMI ≥ 25 kg/m^2^ Subsample: 4 nonobese and 6 obese subjects	Bacterial diversity was significantly greater in obese subjects compared with nonobese subjects. Reduced numbers of Bacteroidetes and a higher F/B ratio in obese subjects compared with nonobese subjects.
	Microbiota fecal samples			
	16S DNA sequencing Metagenome@KIN software			
	Corresponding OTU identified according to T-RFLP			

Million et al. 2012 [23]	Cross-sectional study	115	68 obese and 47 controls	*L*. *reuteri* was associated with obesity. *M*. *smithii* was depleted in obese subjects. Some *Bifidobacterium* or *Lactobacillus* species were associated with normal weight (*B*. *animalis*).
	Microbiota fecal samples			
	qPCR targeting Firmicutes, Bacteroidetes, *Lactococcus lactis*, *Methanobrevibacter smithii*, and *Bifidobacterium animalis*			

Haro et al. 2016 [24]	Cross-sectional study	75	39 men and 36 women with CVD within CORDIOPREV study 3 groups according to BMI: BMI < 30, 30 < BMI < 33, and BMI > 33	F/B ratio changed with the BMI and between genders. Men had higher F/B ratio under a BMI of 33. By contrast, men had a significantly lower F/B ratio than women in the BMI > 33 group. At genera level, BMI > 33: higher *Bacteroides* genus in women, but decrease in men.
	Baseline fecal samples			
	16S rRNA sequencing			
	QIIME software			

Lin et al. 2015 [25]	Cross-sectional study	659	Healthy Chinese adults Asian: normal BMI < 23 (*N* = 281), overweight 23-<27.5 (*N* = 304), and obese > 27.5 (*N* = 55)	BMI was not associated with the bacterial community diversity as assessed by alpha diversity in the models.
	Upper gastrointestinal microbial diversity			
	16S rRNA sequencing			
	HOMIM software			

Angelakis et al. 2015 [26]	Cross-sectional study	10	5 lean subjects: BMI 20.7 5 obese subjects: BMI 36.8	Firmicutes and Actinobacteria were the most predominant phyla of the bacterial composition of the duodenal microbiota in both groups. The obese group presented a higher proportion of anaerobic genera and a lesser proportion of aerobic genera, mostly associated with the presence of *Veillonella*, *Bulleidia*, and *Oribacterium*.
	Duodenal microbiota			
	16S rDNA sequencing			
	Illumina MiSeq			

Finucane et al. 2014 [27]	Review of 4 different studies Human Microbiome Project (HMP) and MetaHIT	159	HMP project: 24 obese (BMI > 30) and 123 lean (BMI < 25) individuals MetaHIT project: Danish MetaHIT cohort included 12 individuals (BMI > 35)	The interstudy variability in the taxonomic composition of stool microbiomes far exceeds differences between lean and obese individuals within studies. No quantitative association between the continuous BMI variable and the ratio of B/F. Variation in the relative abundance of F and B is much larger among studies than between lean and obese individuals within any study. MetaHIT and HMP go in the opposite direction [11].

Goodrich et al. 2014 [30]	Cross-sectional study	977	Twin population: 416 twin pairs, mostly females, mean age 60.6 ± 0.3 years *N* = 433: BMI < 25 *N* = 322: BMI 25–30 *N* = 183: BMI > 30	The family Christensenellaceae was significantly enriched in subjects with a BMI < 25 compared to those with BMI > 30. Overall, a majority (*n* = 35) of the OTUs with highest heritability scores were enriched in the lean subjects. A subset of OTUs classified as *Oscillospira* were enriched in lean subjects, and *M*. *smithii*, though not significantly heritable, was positively associated with a lean BMI.
	Fecal samples from the twins UK population			
	16S rRNA			
	Illumina MiSeq			
	QIIME software			

Bondia-Pons et al. [35]	Cross-sectional study	50	16 healthy monozygotic twin pairs discordant for weight (BMI difference > 3 kg/m^2^) Control pairs: nine concordant monozygotic pairs	No differences in fecal bacterial diversity were detected when comparing cotwins discordant for weight. We found that within-pair similarity is a dominant factor in the metabolic postprandial response, independent of acquired obesity.
	Fecal samples			
	Diversity of the major bacterial groups by using 5 different validated bacterial group-specific DGGE methods			

Murugesan et al. [31]	Cross-sectional study	190	190 unrelated Mexican children 9–11 years old 81 normal 29 overweight 80 obese	No statistical significant differences in abundance of phylum.

Ignacio et al. [32]	Cross-sectional study	84	30 obese, 24 overweight, and 30 lean children (3–11 years old)	*B*. *fragilis* group and *Lactobacillus* spp. were found at high concentrations in obese and overweight children when compared with the lean ones and positively correlated with BMI. *Bifidobacterium* spp. were found in higher numbers in the lean group than the overweight and obese ones. Furthermore, a negative correlation between BMI and *Bifidobacterium* spp. copy number was observed.

Hu et al. [33]	Cross-sectional study fecal samples from 67 obese (BMI > 30 kg/m^2^) and 67 normal (BMI < 25 kg/m^2^) individuals	134	Korean adolescents aged 13–16 years	No significant differences in the Bacteroidetes, Firmicutes, and Proteobacteria populations in samples from normal and obese adolescents at the phylum level, although the proportion of *Bacteroides* was highest in normal children (45%), whereas that in obese was 25%. Conversely, the proportion of *Prevotella* in BMI < 25 was 16%; obese adolescents (35%).

T-RFLP reference human fecal microbiota profiling; qPCR: quantitative PCR; CVD: cardiovascular disease; DGGE: denaturing gradient gel electrophoresis.

**Table 2:** Bariatric surgery clinical trials.

Study identification	Description	*N*	Population description	Outcomes
Palleja et al. 2016 [36]	Longitudinal observational study	13	Participants were recruited for bariatric surgery: BMI > 40 kg/m^2^ or BMI > 35 kg/m^2^ with T2D/hypertension	Gut microbial diversity increased within the first 3 months after RYGB and remained high 1 year later. RYGB led to altered relative abundances of 31 species: *Escherichia coli*, *Klebsiella pneumoniae*, *Veillonella* spp., *Streptococcus* spp., *Alistipes* spp., and *Akkermansia muciniphila*.
	Fecal samples			
	Quantification of gut microbiomes at baseline (*n* = 13), 3 months (*n* = 12) after RYGB, and 12 months (*n* = 8) after RYGB			
	16S rDNA shotgun sequencing			

Tremaroli et al. 2015 [29]	Clinical trial	21	Weight-stable women 9 years after randomization to either RYGB or LSG and matched for weight and fat mass loss Control: 2 groups of nonoperated women with BMI matched to patients' (1) presurgical BMI and (2) postsurgical BMI	Significant differences in microbiota composition for RYGB versus OBS samples but not for LSG versus OBS or RYGB versus LSG. 29 microbial genera differed significantly between RYGB and controls: ↑ Gammaproteobacteria class and ↓ three species in the Firmicutes phylum (*C*. *difficile*, *C*. *hiranonis*, and *G*. *sanguinis*). At the genus level: ↑ Proteobacteria (*Escherichia*, *Klebsiella*, and *Pseudomonas*) in RYGB. No different microbiota profiles for RYGB and LSG patients.
	Fecal samples			
	16S rDNA			
	Illumina HiSeq 2000			
	Shotgun sequencing			

Damms-Machado et al. 2015 [34]	Clinical trial	10	10 unrelated subjects with obesity grade III at 3 time points: (1) baseline (2) 3 months after LSG (3) 6 months after LSG (*N* = 5) or dietary weight loss regimen (*N* = 5)	Both interventions resulted in changes of the B/F ratio but with an inverse relationship between the main phyla. LSG: ↑ Bacteroidetes and ↓Firmicutes Dietary intervention: ↓ Bacteroidetes and ↑Firmicutes In LSG: Bacteroidetes correlated negatively with weight. Firmicutes positively correlated with weight.
	Fecal samples			
	SOLiD long-mate-paired shotgun sequencing			

Graessler et al. 2013 [37]	Clinical trial	6	3 men and 3 women, recruited for RYGB 38–53 years Preoperative BMI 40.9–52.1	↓ Firmicutes, Bacteroidetes, Actinobacteria, and Cyanobacteria. However, the ratios of B/F shifted from 0.99 to 1.31, showing an apparent increase. ↑ Proteobacteria, Verrucomicrobia, and Fusobacteria. 3 months after RYGB, ↑ *E*. *cancerogenus*, *Veillonella parvula*, *V*. *dispar*, *Shigella boydii*, and *Salmonella entérica*. Postoperative abundances of these species were also significantly higher than those in lean controls.
	Fecal samples			
	16S rDNA			
	Illumina HiSeq 2000			
	Shotgun sequencing			

BMI: body mass index, expressed as kg/m^2^; RYGB: Roux-en-Y gastric bypass; LSG: sleeve gastrectomy.

## Supplementary Materials

Supplementary Materials Figure 1: PRISMA-based flowchart of all the records searched.
