# Supplementary material for: The Gut Microbiome Profile in Obesity: A Systematic Review
Source: Int J Endocrinol. 2018 Mar 22;2018:4095789. doi: 10.1155/2018/4095789 (PMC5933040; doi:10.1155/2018/4095789)
Supplement: Supplementary Materials — Figure 1: PRISMA-based flowchart of all the records searched. [file 4095789.f1.pdf]

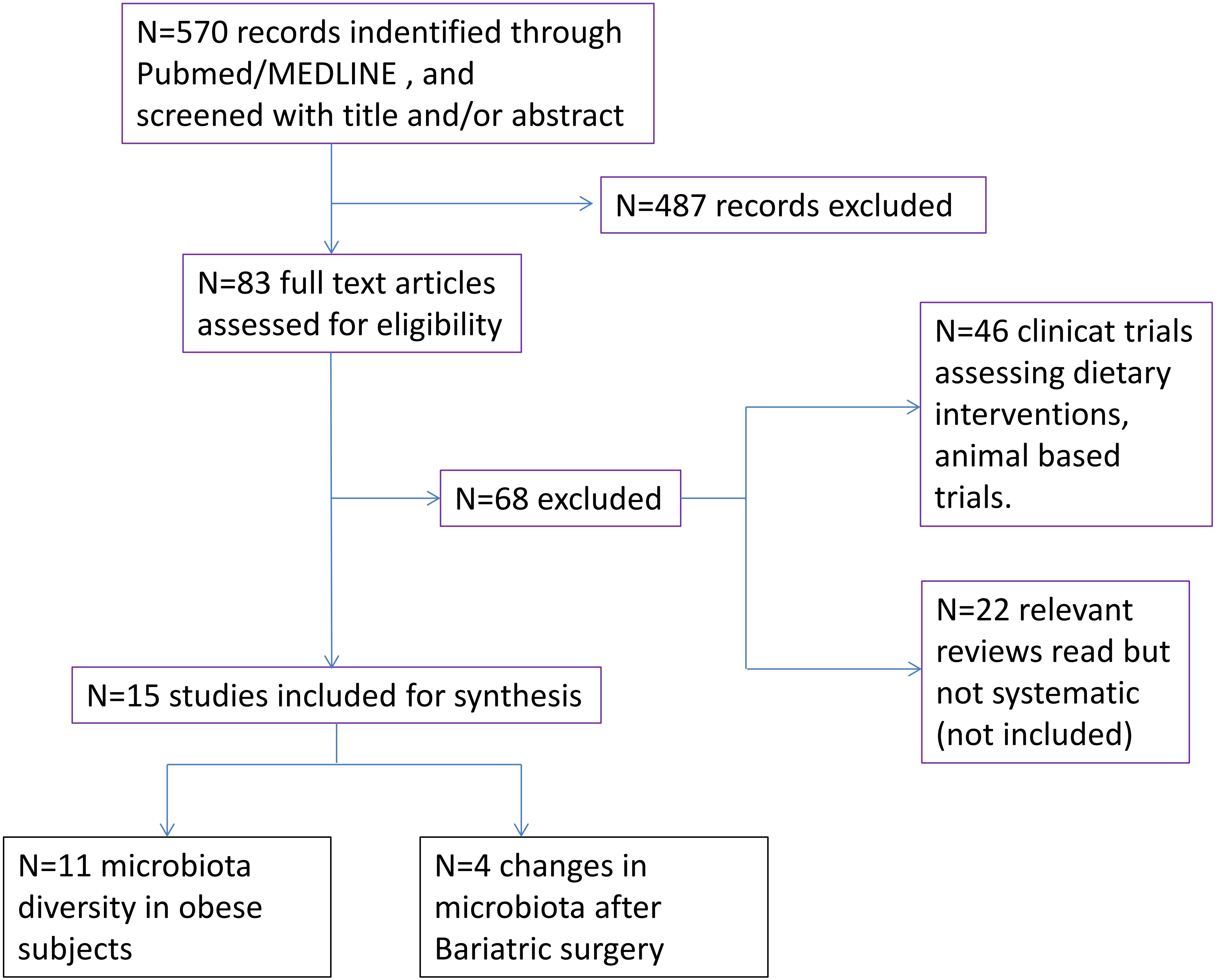
N=570 records indentified through Pubmed/MEDLINE , and screened with title and/or abstract
N=487 records excluded
N=83 full text articles assessed for eligibility
N=68 excluded
N=46 clinicat trials assessing dietary interventions, animal based trials.
N=22 relevant reviews read but not systematic (not included)
N=15 studies included for synthesis
N=11 microbiota diversity in obese subjects
N=4 changes in microbiota after Bariatric surgery
